# ORF Capture-Seq as a versatile method for targeted identification of full-length isoforms

**DOI:** 10.1038/s41467-020-16174-z

**Published:** 2020-05-11

**Authors:** Gloria M. Sheynkman, Katharine S. Tuttle, Florent Laval, Elizabeth Tseng, Jason G. Underwood, Liang Yu, Da Dong, Melissa L. Smith, Robert Sebra, Luc Willems, Tong Hao, Michael A. Calderwood, David E. Hill, Marc Vidal

**Affiliations:** 1 Center for Cancer Systems Biology (CCSB), Dana-Farber Cancer Institute, Boston, MA 02215 USA; 2 Department of Genetics, Blavatnik Institute, Harvard Medical School, Boston, MA 02115 USA; 3 Department of Cancer Biology, Dana-Farber Cancer Institute, Boston, MA 02215 USA; 4 Department of Biochemistry, Northeastern University, Boston, MA 02115 USA; 5 Department of Genetics and Genomic Sciences, Icahn School of Medicine at Mount Sinai, New York, NY 10029 USA; 6 Icahn Institute of Data Science and Genomic Technology, New York, NY 10029 USA; 7 Laboratory of Molecular Biology, TERRA Teaching and Research Centre, Gembloux Agro-Bio Tech, University of Liège, Gembloux, 5030 Belgium; 8 Laboratory of Molecular and Cellular Epigenetics, GIGA-Cancer, University of Liège, 4000 Liège, Belgium; 9 Pacific Biosciences, Menlo Park, CA 94025 USA; 10 School of Computer Science and Technology, Xidian University, Xi’an, 710071 China

**Keywords:** Genomics, DNA sequencing, RNA sequencing, RNA splicing

## Abstract

Most human protein-coding genes are expressed as multiple isoforms, which greatly expands the functional repertoire of the encoded proteome. While at least one reliable open reading frame (ORF) model has been assigned for every coding gene, the majority of alternative isoforms remains uncharacterized due to (i) vast differences of overall levels between different isoforms expressed from common genes, and (ii) the difficulty of obtaining full-length transcript sequences. Here, we present ORF Capture-Seq (OCS), a flexible method that addresses both challenges for targeted full-length isoform sequencing applications using collections of cloned ORFs as probes. As a proof-of-concept, we show that an OCS pipeline focused on genes coding for transcription factors increases isoform detection by an order of magnitude when compared to unenriched samples. In short, OCS enables rapid discovery of isoforms from custom-selected genes and will accelerate mapping of the human transcriptome.

## Abstract (web summary)

Most human protein-coding genes are expressed as multiple isoforms. Here the authors present ORF Capture-seq that uses cloned ORFs as probes to capture and sequence full length transcript sequences. This enables highly sensitive characterization of eukaryotic transcriptomes.

## Introduction

Mechanisms that enable production of multiple isoforms from a single gene—such as alternative transcriptional start sites, splicing, and polyadenylation—contribute to expanding the functional capacity of the encoded proteome^1–3^. The full extent of this capacity is unknown, as we are currently unable to generate an accurate and comprehensive catalog of the human transcriptome^4^. Although advances in high-throughput sequencing have enabled mapping of local elements (e.g., individual splice junctions), how these elements combine to form full-length isoforms is largely unknown. Short-read RNA-seq data from currently popular platforms (<250 bp, Illumina) fail to resolve such sequences^5,6^. Consequently, the majority of annotated isoform models remain as predictions derived from partial transcripts, particularly for context-specific, disease-specific, or low abundance isoforms^4,7^.

Long-read sequencing platforms—PacBio^8^, Oxford Nanopore^9^, and those based on adaptations to next generation sequencing that produce synthetic long reads such as 10X and Moleculo^10^ sequencing—can return unambiguous, full-length isoform sequences that fully resolve transcriptome complexity. However, in comparison to short-read sequencing platforms, these methods suffer from lower sampling sensitivity and can miss low (<10 copies/cell) to moderate (10–50 copies/cell) abundance transcripts^11^. A number of transcripts at these abundance levels are responsible for producing disease-associated or important regulatory proteins (e.g., transcription factors and kinases)^12^. The sensitivity problem is exacerbated by the wide dynamic range of the human transcriptome across at least six orders of magnitude^13^, causing an inordinate amount of sequencing effort to be used for detecting the most abundant isoforms. Therefore, most transcripts of lower abundance are not sequenced satisfactorily due to this biased sampling.

An established solution to increase detectability of isoforms is targeted sequencing, which involves enriching for transcripts of desired genes. DNA or RNA hybridization-based enrichment followed by high-throughput sequencing is a particularly efficient, robust, and cost-effective solution^14^. These approaches were initially developed for targeted sequencing of protein-coding regions of genomic DNA (i.e., whole exome sequencing)^15^ and RNA fragments from short-read RNA-seq experiments (e.g., CaptureSeq)^16–20^. Such approaches have been adapted for targeted sequencing of long genomic fragments (>2 kb) or full-length cDNA molecules^21–26^. In two notable studies described recently, complex pools of biotinylated oligos are used to enrich for full-length cDNA corresponding to thousands of protein-coding and non-coding RNA targets. The enriched material is subjected to long-read sequencing, leading to considerable gains in full-length isoform detection and insights about the nature of transcriptomic complexity^27,28^.

The success of these targeted full-length sequencing methods hints at the potential of using this approach in a more general framework. Previous studies employ a single panel of probes with many targets. This schema worked well for exome sequencing, in which a single probe set designed against all protein-coding exons yields high coverage of all DNA targets, each of which are present at identical concentration (2 copies/cell). However, such high coverage is challenging to attain from transcriptome sequencing, because transcriptomes are highly heterogeneous, with a wide dynamic range and variation in composition (i.e., set of genes expressed), depending on cellular or disease contexts. Therefore, a single probe set enables increased sequence sampling of isoforms from genes of interest, but the expression patterns within the genes of interest will still be skewed, reducing coverage. It is thus essential to devise a flexible strategy to generate with ease multiple, distinct probe sets that match the particular transcriptome context, specifically, to enable facile detection of isoforms from any set of genes from any set of samples.

Here we present ORF Capture-Seq (OCS), a generalizable method for direct synthesis of biotinylated capture oligos from existing or newly designed ORF clones followed by targeted enrichment and sequencing of full-length cDNAs to discover new isoforms. The unique combination of low cost, time, ease, and versatility (any pool of ORFs or clones, up to thousands at-a-time) of the method offers the experimental flexibility needed to rapidly characterize any desired subset of the transcriptome. Using reagents and instruments available in most molecular biology laboratories, a user can synthesize probes from one or a set of amplicons or clones in less than 24 h. We envision this method will be of broad utility in many applications, from single-gene studies to system-scale applications seeking to characterize whole transcriptomes. Here, we compare OCS probes against a commercial standard, benchmark the method using spike-in standards, and apply it towards characterization of novel isoforms of ~800 human transcription factors (TFs).

## Results

### OCS method for flexible targeted sequencing

**Fig. 1:**
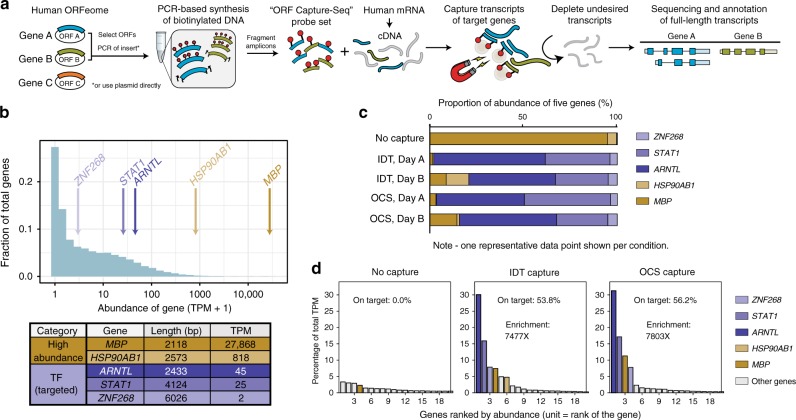
ORF Capture-Seq (OCS) method for accelerated discovery of full-length isoforms. **a** Schematic of the OCS method. ORF clones of target genes are pooled and used as templates for a biotin-dUTP-labeling PCR reaction, creating randomly biotinylated amplicons which are fragmented to generate a probe set. In this study, PCR-based amplicons derived from the clones were used as template. These OCS probes can be used in targeted sequencing applications, such as enrichment of full-length cDNA for sequencing on the PacBio platform. **b** Transcriptional abundances in human brain cDNA. These values were used as the basis for selecting three low to moderate abundance transcription factors (TFs) as target genes (purple labels) and two high abundance genes (yellow labels) as background controls. Length is an average of all transcripts annotated for each gene (GENCODE v22). TPM values were obtained from processing Illumina sequencing data (Methods). TPM, transcripts per million. **c** Comparison of IDT vs OCS-based target enrichment. Each bar shows the relative proportion of cDNA from target (purple) versus background (yellow) genes as quantified by qPCR (average of two technical replicates). A total of three individual capture reactions were performed per day (see Supplementary Fig. 1e for full dataset) over two days (Day A, B). Only one of the three reactions is shown in this figure. **d** Individual gene expression, ranked in descending abundance, as quantified by Illumina sequencing and Kallisto (Methods). Each bar represents one gene. Only the 20 most abundant genes are shown. Bars are color coded as background controls (yellow), target genes (purple), and all remaining genes that were not targeted (gray). On-target percentages are the fraction of transcriptional abundance corresponding to the three targeted TFs (*ARNTL*, *STAT1*, *ZNF268*), in each capturant. Fold enrichment is computed by dividing percentage of targets in the capturant by the percentage in the unenriched input. Source data are available in the Source Data file.

The OCS pipeline begins with flexible and straightforward synthesis of biotinylated capture probes (Fig. 1a). PCR is performed on any number of pooled templates (e.g., plasmids, amplicons) using universal primers in the presence of biotin-dUTP. The resulting pool of biotinylated PCR products, with biotin-dUMP incorporated throughout, are randomly sheared into overlapping DNA fragments of ~150 bp. This generates a set of overlapping fragments from each PCR amplicon. After purification and removal of PCR primers and unincorporated nucleotides, the resulting OCS probe set is used for hybridization-based capture of target nucleotide sequences.

We demonstrate the application of OCS for enrichment and sequencing of full-length transcripts from protein-coding genes, though the method can also be applied to non-coding RNAs as well (Fig. 1a). Probes are derived from one or more ORF(s) or PCR amplicon(s). Though each ORF represents just one isoform of a gene, the corresponding probes are expected to capture all isoforms, due to the high sequence overlap between isoforms of the same gene; probes need to only target a portion of a full-length cDNA for enrichment^18^. We capitalized on the availability of our human ORFeome collection, a resource of freely available Gateway Entry clone ORFs for ~17.5 K of the ~20 K protein-coding genes in human^29^, creating from this resource customized pools of ORF clones to use as templates for biotin-labeling PCR. All clones share a common vector backbone, enabling production of any amplicon from universal primers. Tens to hundreds to thousands of ORF clones may be pooled and processed together so that complex and customized probe sets can be generated with relative ease.

We emphasize that though the demonstration of OCS in this paper involves a large-scale application where templates are derived from a comprehensive ORFeome collection, our OCS method is highly applicable to smaller-scale studies using a small number of clones or amplicons.

### OCS probes perform comparably to a commercial standard

We first established that OCS probes are comparable to commercially synthesized biotinylated probes in terms of enrichment efficiency.

To benchmark, we selected three low abundance human TF genes (*ARNTL*, *STAT1*, and *ZNF268*) expressed in brain. The enrichment of these TFs was compared against two high abundance housekeeping genes (*MBP* and *HSP90A1*) to serve as a measure of off-target binding (Fig. 1b). For each of the three TFs, OCS and commercial probes were synthesized (Supplementary Fig. 1a). Probes were not generated for the two housekeeping genes. OCS probes were sequenced on an Illumina MiSeq, confirming an even distribution of probe coverage (estimated ~150X tiling density) and high purity (Supplementary Fig. 1b–d). Commercially available probes were synthesized as 5’ biotinylated 120-mers with a ~1X tiling density against both the forward and reverse strands (Supplementary Fig. 1a, Supplementary Table 1) by Integrated DNA Technologies (IDT).

We compared OCS and IDT probes for ability to enrich for transcripts from the three TF genes in human brain cDNA and found them to be comparable. Based on qPCR against target and housekeeping genes, both probe sets produced ~80% on-target rate with similar degrees of technical variability (Fig. 1c, Supplementary Fig. 1e). Importantly, the on-target enrichment rates, defined as the fold increase in relative abundances of the TFs, were statistically indistinguishable between OCS and IDT probes (Supplementary Fig. 1f). A capture reaction employing OCS probes from a second independent synthesis exhibited consistent performance (Supplementary Fig. 1g). We then measured on-target rate by sequencing a subset of the technical replicates using RNA-seq (Illumina MiSeq, see Methods) and estimated an on-target rate of 54% for OCS and 56% for IDT (Fig. 1d).

A possible concern with using OCS probes, which are derived from PCR inserts in which each ORF is flanked by ~100–150 bp of vector backbone^29^, is that probe sequences arising from the vector sequences can cause non-specific binding. To investigate this, we compared background binding profiles derived from OCS versus IDT capture experiments. The profiles are displayed as enrichment of each transcript as a function of initial abundance, because higher abundance transcripts have been observed to non-specifically bind to the beads (i.e., streptavidin beads which bind to and enrich probe-target complexes) to a greater extent than low abundance transcripts (Supplementary Fig. 1h, see *No Probe Control*). We found no systematic bias using either probe type.

### Analytical benchmarking of OCS using spike-in standards

Next, we benchmarked the analytical performance of OCS by employing External RNA Controls Consortium (ERCC) standards, which are 92 synthetic ORFs of concentrations spanning 10-orders of magnitude^30^.

**Fig. 2:**
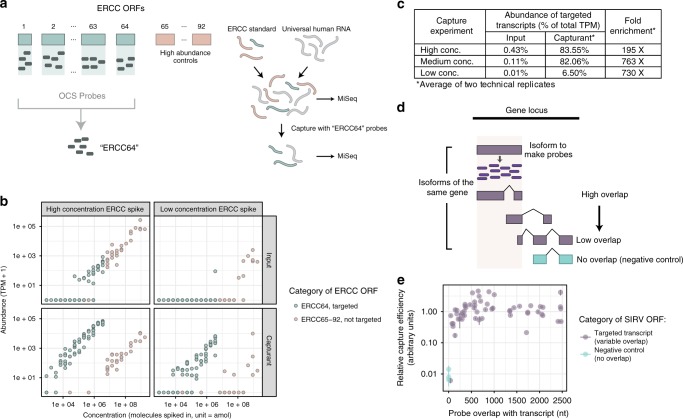
Benchmarking OCS analytical performance. **a** Schematic of benchmarking experiment using ERCC standards. **b** Enrichment of ERCC targets. The *x*-axis represents the nominal concentration of ERCC RNAs spiked into the starting pool of RNA (input) and the *y*-axis represents estimated abundance of each ORF in the input cDNA (top row) or capturant (bottom row). Each point represents a distinct ERCC standard (92 in total) which was targeted (light blue) or not targeted (pink). **c** Summary statistics related to capture efficiencies for ERCC capture reactions. **d** Schematic of probe synthesis using the SIRV system. **e** Relationship between enrichment efficiencies at different isoform overlaps. The isoform overlap represents the absolute number of nucleotides overlapping between (i) the template isoform used to generate probes, and (ii) the target isoform present in the sample. Negative controls are non-overlapping isoforms. Capture efficiencies were computed by dividing read depth of each SIRV (isoform) in capturant by the read depth in input cDNA. Error bars, standard deviation. Source data are available in the Source Data file.

To assess specificity and reproducibility, we measured enrichment of a subset of ERCC ORFs in human reference RNA (Fig. 2a). OCS probes were synthesized for the 64 ERCC ORFs of lowest concentration (*ERCC64*, Supplementary Fig. 2a, Supplementary Table 2,). The remaining 28 ORFs of highest concentration were not targeted and served as controls. ERCC RNA standards were spiked into Universal Human Reference RNA (UHRR) at high (1:10, i.e., 10X dilution of ERCC standard), medium (1:80), and low (1:5120) concentrations, in technical duplicates (Supplementary Fig. 2b). Input cDNA and capturant cDNA were sequenced on an Illumina MiSeq and abundance values estimated using the Kallisto software package^31^, in which a subsampling of 100 K reads were subjected to analysis to allow for comparison of ORF detection at comparable sequencing depth.

We show that OCS probes successfully enriched all 64 targeted ERCCs at uniformly elevated levels (Fig. 2b). We computed overall on-target rates by mapping reads to a reference transcriptome (GENCODE) and computing the total abundance, in transcripts per million (TPM), arising from ORFs targeted for enrichment. Fold enrichment was calculated by dividing on-target percentages detected in the capturant by those found in the unenriched input. For the high- and medium-spike capture, on-target rates were above 80% with fold enrichments of 195- and 763-fold (Fig. 2c). For the low-spike capture, on-target rates were lower (~6%) but enrichment was high (730-fold). We observed that the relative abundances of targeted ERCCs remained linear post-capture, indicating potential for quantification as long as probes are in excess concentration compared to the target cDNA (Supplementary Fig. 2c, see Clark et al. for more details^32^). Furthermore, though the correlation passed statistical significance, the extent of enrichment was not markedly affected by properties such as starting concentration, GC content, ORF length, and probe representation (Supplementary Fig. 2d). Importantly, we did not detect a significant drop in enrichment efficiency for longer ORFs (up to ~2 kb) nor ORFs with lower probe representation, as may be expected. Technical reproducibility was also high, with Pearson’s *R*^2^ above 0.97 for all technical replicates (Supplementary Fig. 2e).

### OCS enriches a family of isoforms using only one isoform

The use of OCS for isoform discovery relies on the assumption that probes derived from a single isoform can efficiently enrich for all isoforms of a given gene. Indeed, there is typically sufficient overlap between any given isoform sequence and all other isoforms of that gene. However, to address the concern that there could be lower capture efficiency due to low sequence overlap, we measured the relationship between overlap and capture efficiency.

For this purpose, we used Spike-In RNA Variant (SIRV) standards (Lexogen), consisting of 69 synthetic isoforms with highly complex splicing patterns from seven artificial genes (Fig. 2d)^33^. We synthesized OCS probes from one representative SIRV isoform per gene (*SIRV7*, Supplementary Fig. 2f, g, Supplementary Table 3) and used this probe set to enrich for all SIRV isoforms that were spiked into UHRR (Supplementary Fig. 2h). We found no appreciable difference in capture efficiency when sequence overlap ranged between 45 and 2500 nt (Fig. 2e). Only in one extreme case, where overlap was only 35 nt, did capture efficiency sharply decline to the level of negative controls. These results are consistent with our observation that captures employing a single probe have good enrichment. For example, use of a single probe from the *ARNTL* IDT probes enabled large enrichments of *ARNTL* isoforms (0% in input cDNA, 8 and 10% in the capturant).

Based on the SIRV results, we calculated how well the OCS approach could cover the human transcriptome. Using GENCODE-annotated transcripts, we calculated the degree of overlap between a single representative isoform and all other annotated isoforms of the same gene (Supplementary Fig. 2i, see “Methods” section, Isoform Overlap Estimation). The overlap between pairs of isoforms (one representative versus all isoforms of a gene) was calculated by taking the intersection of genomic ranges. If the representative isoform is set to the principle isoform in the APPRIS database, as defined by GENCODE^34^ (version 29), we estimate that 99.7% of all isoforms are potentially captured by OCS probes designed against the principal isoform (overlap of 50 bp or more, based on results from the previous SIRV). If the representative isoform was set to a randomly chosen isoform, we estimate that 99.3% of all isoforms are covered. The slight decrease in coverage can be explained by the fact that that the APPRIS principle isoform tends to be longer in length than randomly chosen isoforms, and longer isoforms would more likely overlap other isoforms of that gene.

### Applying OCS to characterize human TF isoforms

Alternative transcriptional start sites, splicing, and polyadenylation can modulate the activity of TFs by altering sequences corresponding to DNA binding, co-factor binding, and other properties such as availability of phosphorylation sites^35,36^. Despite being heavily studied, many TF isoforms remain uncharacterized due to low abundance (<10 copies/cell), complex splicing patterns, or expression in cell-, tissue-, or disease-specific contexts^35–37^. Here, we applied OCS to characterize alternative isoforms of human TFs.

First, we sought to explore the relationship between the number of genes targeted and sensitivity. We synthesized OCS probe sets for 2, 12, 88, and 682 randomly chosen TF genes and applied it to human cDNA derived from cerebral cortex (Supplementary Fig. 3a, Supplementary Tables 4–6, Methods). To unambiguously distinguish different TF isoforms, we subjected the enriched cDNA to long read sequencing in addition to short read RNA-seq.

**Fig. 3:**
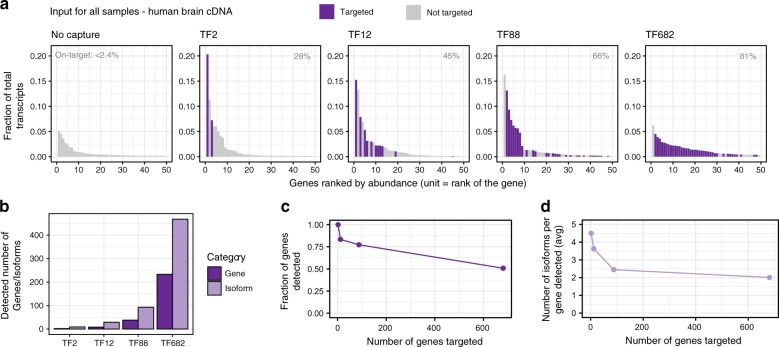
Multiplexing parameters for enrichment of human transcription factors. **a** Rank abundance bar plots for unenriched (input) and enriched (capturant) cDNA. Data is shown for (i) input brain cDNA, and (ii) the series of capturants prepared using probe sets with increasing number of TF genes. Only the top 50 most abundantly expressed genes, calculated per sample, are shown. Each bar corresponds to a single gene, colored by whether that gene is targeted (purple) or not targeted (gray). Fraction of total transcripts was calculated by dividing the transcript abundance (TPM) of all transcripts from a gene by the total transcript abundances for the sample (Methods). On-target rates, as calculated for the entire sample, are displayed on the upper right-hand position of the plots. **b** Absolute number of targeted genes (dark purple) and isoforms (light purple) detected from each capture reaction. **c** Relationship between the number of genes multiplexed and the fraction of genes for which there was a detected full-length read. **d** As in **c** except shows the decrease in isoforms per targeted gene, on average, for each experiment. Source data are available in the Source Data file.

We were able to maintain high capture efficiency even at increased probe set complexity (Fig. 3a, b). However, the limiting sequencing depth reduced the number of genes, as well as isoforms per gene, that were detected (Fig. 3c, d). This was further shown by saturation-discovery curve analysis in which we plotted the number of unique genes and isoforms detectable at different sequencing depths. At 20,000 full-length reads, we reached saturation for the TF2 and TF12 probe sets, but were still discovering new isoforms for the TF88 and TF682 (Supplementary Fig. 3b, Methods). Our results show that additional sequencing is required to reach saturation using these more complex probe sets. We note that, as of this writing, emerging platforms (e.g., PacBio Sequel II) provides much higher throughput than the older platforms (e.g., PacBio RS II, Sequel) used in this study. With increased throughput, there is a potential to saturate the samples at even the isoform-level and also increase the level of sample-multiplexing, further reducing the cost for full-length transcript studies.

### Discovery of isoforms in tissue using transcript sequencing

**Fig. 4:**
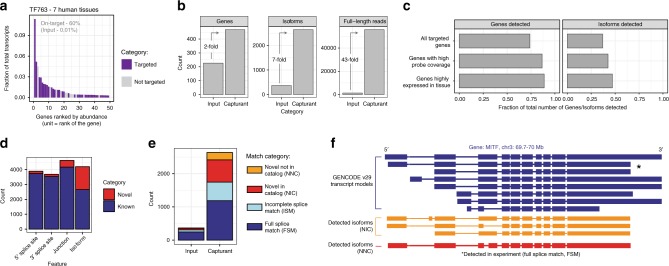
Full-length transcription factor isoforms across diverse human tissues. **a** Rank abundance bar plot for cDNA enriched for human TFs. Only the top 50 most abundantly expressed genes are shown. Each bar corresponds to a single gene, colored by whether that gene is targeted (purple) or not targeted (gray). Abundances as shown on the *y*-axis are computed by dividing the number of full-length reads mapped to the gene by the total number of full-length reads. On-target rates for input and capturant are shown. **b** Gains in coverage of target genes upon enrichment. Increases in number of genes, isoforms, and full-length reads are shown. Data used to generate these numbers used an equal number of full-length raw reads that were subsampled from the unenriched (input) or enriched (capturant) cDNA. **c** Fraction of all GENCODE genes and transcripts detected in the capturant. A gene is considered detected if at least one full-length read is detected for that locus. Isoforms are considered detected if the full set of junctions are identical between the GENCODE-annotated and sequenced transcript. The fraction detected was also computed for sets of genes for which there was higher probe set representation of the gene (1 TPM or higher) and genes for which there was evidence of expression in the tissues interrogated (10 TPM or higher). **d** Fraction of novel splice sites, junctions, and full-length isoforms in the TF enrichment experiment. Unique splice sites and junctions are only counted once. The 5′ splice site corresponds to the splice donor and the 3′ splice site corresponds to the splice acceptor. SS, splice site. **e** Proportions of known and novel isoforms. Known isoforms are further divided by completeness. Novel isoforms are further divided by whether all splice sites are found in GENCODE (novel in catalog, NIC) or if the isoform contains a novel splice site (novel not in catalog, NNC). Match categories are defined by the isoform annotation tool SQANTI. **g** Example of isoforms from the gene *MITF* identified from the TF763 capture experiment. Source data are available in the Source Data file.

We applied OCS towards discovering novel TF isoforms in a diverse set of human tissues. We created an OCS probe set consisting of 763 randomly chosen TFs to enrich from a pool of barcoded cDNA libraries stemming from 7 distinct human tissues -- adult brain, fetal brain, heart, liver, pancreas, placenta, and testes (Supplementary Fig. 4a, Supplementary Tables 7 and 8, Methods). We sequenced the unenriched and enriched cDNA on the PacBio Sequel platform, resulting in 118,872 and 476,589 full-length reads, respectively. Raw data was processed using the Iso-Seq v3 bioinformatic pipeline^38^ followed by SQANTI for isoform annotations^39^. The on-target rate for the capturant sample was ~60%, with most of the top ranked genes being the target TFs (Fig. 4a).

To compare enrichment efficiency, ~100 K full-length reads were sampled from both the input and capturant datasets and sequencing statistics were calculated. We found that the number of genes, isoforms, and full-length reads increased 2-, 7-, and 43-fold after enrichment, respectively, emphasizing the need for enrichment to fully sample the isoform space (Fig. 4b). In theory, if sequencing depth is increased to appropriate levels, any low abundance isoform should be detectable from unenriched cDNA. However, in this case, approximately 40 times more coverage using bulk long-read sequencing would have been needed to attain the coverage achieved using OCS, showing the benefit of target enrichment strategies.

We analyzed the extent to which we recovered full-length isoforms that are annotated in GENCODE. Overall, the recovery of GENCODE genes was 74%, increasing to 86% or higher when considering only genes which were well-represented in the probe set (1 TPM or higher), or genes well-expressed (10 TPM or higher) in the 7 tissues (Fig. 4c, Supplementary Table 9). The recovery of GENCODE isoforms was 37%, also increasing when accounting for probe representation or tissue expression. Note, however, that given the limited tissues analyzed in this study, 100% GENCODE isoform recovery is not expected.

In GENCODE annotations, every isoform is tagged with a transcript support level (TSL) 1-5, denoting the extent of experimental information underlying each isoform model. Isoforms with a TSL of 1 contain high-quality experimental, full-length mRNA sequence support. Isoforms with a TSL of 5 are computational predictions with no experimental support. The full-length sequencing data provided confirmation of 398 GENCODE isoforms of TSL 2-5, which includes 85 computationally predicted isoforms (TSL 5) (Supplementary Fig. 4b, Supplementary Table 10). Thus, OCS is an invaluable tool for confirming isoform models in gene annotations.

We found that OCS-based enrichment not only enables significant increases in detection of annotated isoforms, but discovery of novel isoforms as well. To increase the confidence of novel isoforms found in the TF763 dataset, we employed an orthogonal sequencing approach to validate any novel junctions. We subjected the original RNA to dication-catalyzed RNA fragmentation and random hexamer priming to generate cDNA fragments which were subjected to Illumina sequencing. This random hexamer approach for short-read sequencing, which is not subject to artifacts that come from full-length cDNA amplification, provides orthogonal support for individual junctions found in the long-read data^39^. We required each novel junction found in the PacBio isoforms to be supported by at least three short reads, resulting in a population of novel isoforms with quality features (e.g., non-canonical junction rate), intrinsic sequencing properties (e.g., number of predicted RT artifacts), and functional genomics evidence (e.g., overlap of 5′ end with CAGE peaks) that are indistinguishable from the high-quality, known isoforms that match GENCODE annotations (Supplementary Fig. 4c-k). All subsequent analyses involved this orthogonally-validated set of novel isoforms (Supplementary Table 11).

One potential concern with the use of high-density OCS probes is the formation of chimeric products during subsequent rounds of PCR, which may lead to the formation of artifactual isoforms. Currently, it is not possible to distinguish de facto novel isoforms from chimeric products formed by stitch-like PCR of partial fragments. To experimentally assess the percentage of novel isoforms that are non-chimeric, we employed a dual barcoding strategy used previously to assess this type of artifact^40^. The strategy includes a cDNA preparation step in which each cDNA molecule is synthesized such that known matching barcodes are appended at the 3′ and 5′ ends prior to whole-cDNA PCR amplification. Using this library, cases in which the barcodes are swapped occur only through formation of chimeric products and can be directly detected in the long read sequencing data. We subjected a dual barcoded library generated from human brain cDNA to enrichment with either IDT probes or OCS probes directed against the same three transcription factor genes, as described in Fig. 1. The libraries were sequenced on a PacBio Sequel II system. When considering all isoforms, the rate of non-chimeric isoforms was 99% for IDT and 98% for OCS. When considering only novel isoforms, 98% of the IDT isoforms and 94% of the OCS isoforms were non-chimeric. Therefore, compared to IDT, OCS captures have a slightly elevated rate of chimeric isoforms. Overall, however, both technologies have very low rates of chimera formation and users may find that the cost and flexibility benefits of the OCS method an advantage.

We classified the isoform set against GENCODE v29. Approximately 4% and 10% of all distinct splice sites and junctions were novel, respectively, but a much higher fraction of isoforms, 37%, were novel (Fig. 4d). This can be explained by the fact that a single local event (e.g., one novel splice site) leads to an entirely distinct transcript, in terms of the full-length sequence. Overall, the total number of isoforms detected dramatically increased upon enrichment and at the same time the relative fraction of novel isoforms increased in proportion (Fig. 4e, Supplementary Fig. 4l). A substantial proportion of the reads arose from novel isoforms, though the number of full-length read counts was slightly lower for novel isoforms as compared to known isoforms (Supplementary Fig. 4m). Overall, 1,528 distinct novel isoform sequences from 306 TF genes were detected. Among the TF genes targeted, at least one novel isoform was found for 39% (306/782). A majority of these isoforms (68%) arise from unique combinations of annotated junctions or splice sites, representing a change in the connectivity of known transcript elements. Most of the remaining isoforms (26%) contained novel donor or acceptor splice sites, effectively creating novel exons. A small fraction (5%) of isoforms contained intron retention events. We show an example for the gene *MITF*, which display isoforms which perfectly match a GENCODE transcript, has novel combinations of junctions, and has novel splice sites (Fig. 4f).

## Discussion

Eukaryotic transcriptomes remain unresolved at full-length resolution, and the extent of transcript diversity is unknown. Recently, targeted full-length sequencing methods, in which pools of biotinylated oligos are used to enrich for full-length cDNAs for sequencing, have characterized focused subsets of the transcriptome with great depth and accuracy^27,28^. Here, we establish that ORF Capture-Seq is a versatile method to synthesize probes that can be used for comprehensive enrichment of cDNAs for targeted full-length sequencing studies. Compared to traditional or microarray-based synthesis of biotinylated oligos, OCS stands as a complementary strategy for synthesis of simple to complex probe sets for desired genes. OCS enables direct characterization of isoform sequences without prior information about transcript boundaries or exons, unlike with RACE- or PCR-based sequencing. This facilitates discoveries of the full array of known and novel isoforms for other classes of proteins (e.g., kinases, G protein-coupled receptors) or targets involved in a biological pathway of interest or implicated in human genetic diseases. Applying OCS to detect TF isoforms in human tissues, we found a preponderance of novel isoforms, detecting over a thousand novel TF isoforms.

Several applications involving OCS can be envisioned. First, isoform expression information could guide high-throughput cloning efforts^41^. Second, OCS can help define full-length isoform sequences from genes exhibiting differential splicing at the local level, using programs like Leafcutter^42^. Third, it also provides opportunities for increased accuracy in isoform quantification workflows. Here, knowledge of isoforms expressed in a sample, as informed by long-read data, can serve as the scaffolds (i.e., gene models) upon which short reads rely to estimate isoform abundances^39,43^. Finally, OCS probes could conceptually be applied at the single cell level, as pools of barcoded single cell cDNA could be subjected to enrichment similarly to bulk cDNA preparations. Indeed, a method using commercial probes to enrich for T-cell-receptor and B-cell-receptor transcripts from single cells was recently described^44^.

The ease of making probes using the OCS method opens doors for novel strategies in capture experiments. For example, a series of captures can be designed in an iterative manner, in which the initial capture returns the first batch of detected genes, and subsequent captures use probe sets that include only genes that failed to return isoforms in the first round. Alternatively, multiple gene panels may be created, stratified by endogenous abundance of genes (e.g., separation of probe sets for low and high abundance transcripts) or priority of disease genes (e.g., low/high confidence of association). Furthermore, the representation of genes within a probe set is customizable to the greatest extent. For example, the concentration of individual ORFs may be titrated based on desired factors, such as priority or endogenous abundance in the sample, so that the relative coverage of isoforms from different genes are normalized, allowing for greater sequencing coverage.

Some limitations of OCS remain. For example, since the probe synthesis step relies on PCR, one constraint of the method lies in the length of ORF clones that can be used as templates. Indeed, while probe synthesis and full-length sequencing for transcripts up to 4 kb is attainable, employing the method for long transcripts above 5–6 kb is a challenge. For longer transcripts, other mechanisms to generate biotinylated probes may be required (e.g., 5′ biotinylated directed^45^ or random primers, ligation^46,47^, or nick translation^48–50^). Alternatively, we generated probes from individual segments of an ultra-long ORF clone (unpublished). Another limitation is some targeted transcript may be missed if there is insufficient overlap between the ORF used for probe synthesis and targeted transcripts (isoforms) of the same gene, although we estimate this will occur only very rarely based on annotated transcripts.

Another challenge is not just related to OCS but all long-read cDNA sequencing methods. Given the high number of novel isoforms routinely detected in long-read sequencing studies, an open question in the field is how to best assess the quality of these isoforms and to understand all sources of artifacts that could arise from the biochemical and analytical preparation of samples for sequencing. For example, the OCS protocol, as well as any targeted long-read method, requires PCR to amplify the enriched cDNA, and a valid concern is the extent of PCR artifact formation. A recent study employed a dual-barcoding approach to find that ~1% of cDNAs sequenced were PCR chimeras (i.e., hybrid molecules from recombinational events during PCR)^40^. In our own study, we found a very low rate of false positive isoform identifications resulting from chimeric reads.

At a more general level, few studies have systematically investigated the sources and frequencies of artifacts. One of the most comprehensive assessments to date is work from Tardaguila and colleagues^39^, in which they evaluated intrinsic and sequence-related properties that contribute to isoform artifacts. They found that non-canonical or RT-template switching junctions underlie poor quality novel isoforms, but that experimental validation using an orthogonal approach effectively detects and removes these events. We employed a similar orthogonal validation in this study. Overall, until full understanding of artifacts is achieved, we recommend that any novel isoform must be viewed as a candidate isoform until it is validated by another assay or combined with strongly supporting functional data. Such evaluation was done for several TF isoform sequences in this study, which led to their acceptance as official transcript models in GENCODE (personal communication with Adam Frankish).

OCS stands as a complementary approach to previously described targeted methods, specifically for synthesizing probes, and delivers unique benefits in certain contexts. Previously described methods for targeted long-read sequencing employed probes that tile across target elements (e.g., genes, predicted lncRNAs) which were designed in silico and synthesized by commercial vendors to generate biotinylated oligo pools. OCS is a viable alternative to generate probes in a flexible manner. For the many laboratories in which researchers have at hand a clone or the ability to generate a PCR amplicon representing a transcript from a gene of interest, probes can be made within 24 hours at nominal cost using widely available molecular biology reagents (Taq polymerase, NTPs, biotin-dUTP, etc.). For larger-scale applications, such as what was demonstrated in this work, some researchers may be interested in investing in a clone collection to capitalize on the ability to rapidly generate probe sets for a near-infinite combination of genes, such as those from a disease pathway. Regardless of the scale, a key benefit is that OCS probes have ultra-high tiling density and can be generated at near inexhaustive supply (e.g., 100+ enrichment reactions per PCR).

We recognize that commercial probes (e.g., IDT probes) could be more useful in certain circumstances, such as when clones are unavailable (e.g., novel lncRNAs) or when targeting predicted genes. Some researchers may prioritize the convenience of direct-to-order commercial probes, without additional labor required to make OCS probes.

In conclusion, OCS is a highly generalizable strategy to synthesize probes for use in full-length capture experiments. Though we demonstrated OCS as applied towards characterization of isoforms originating from protein-coding genes, it can be adapted for use in capture and characterization of different types of genetic and post-transcriptional variants, such as genetic variations, segmental duplications, or lncRNAs. For example, multiple isoforms of lncRNAs are routinely being characterized^28^. It is also possible that ORFs from one species could be used to enrich for isoforms from another species, given high sequence conservation of protein-coding regions (e.g., human ORFs to enrich for mouse isoforms). Overall, we envision this approach will be of broad utility for application within both basic research and the clinical and diagnostic fields^51^.

## Methods

### ORF Capture-Seq probe synthesis

ORF amplicons corresponding to *ARNTL, STAT1, ZNF268* (three TFs) were generated. Our human ORFeome contains one representative ORF, in the form of a Gateway entry clone in pDONR223^52^, for ~17.5 K of the ~20 K human protein-coding genes^29^. These ORF clones are available as bacterial (DH5α) culture glycerol stocks. Bacterial stocks corresponding to the three TFs were cherry-picked from the human ORFeome. Using ~1 µl of culture as template, the ORF inserts were PCR amplified with Platinum Taq DNA Polymerase High Fidelity (Invitrogen) using pDONR223 universal primers with the following sequences:

FOR: CCCAGTCACGACGTTGTAAAACG

REV: GTAACATCAGAGATTTTGAGACAC

PCR was performed for 35 cycles, with each cycle consisting of denaturation at 94 °C for 30 s, annealing at 57 °C for 30 s, and extension at 72 °C for 5 min. The final extension was for 15 min. PCR products were analyzed via agarose gel electrophoresis to confirm that amplicons were of the expected size. Products were purified using Agencourt AMPure XP beads (Beckman Coulter) and quantified using the Qubit dsDNA HS assay (Thermo Fisher Scientific).

A biotin-labeling PCR was done to generate amplicons which contain randomly incorporated biotin-dUTP. Both control and biotin-dUTP PCRs were performed for each TF. Using as template 1 ng of the ORF amplicon, biotin-spiked PCR was done using Taq polymerase (NEB). The dNTP mixture was modified so that a third of the dTTPs were substituted with Biotin-16-Aminoallyl-2′-dUTP (Trilink), referred to heretofore as biotin-dUTP. The program was run for 30 cycles, with each cycle consisting of denaturation at 95 °C for 15 s, annealing at 57 °C for 30 s, and extension at 68 °C for 5 min. The final extension was for 10 min.

The biotin-labeled amplicons were randomly fragmented via sonication. Control and biotinylated amplicons were displayed on an agarose gel to confirm successful PCR. Products were transferred to Covaris AFA FiberCrimp Cap microTUBEs and fragmented on a Covaris E220 sonicator to size distribution of ~150 bp. The sonication method parameters are as follows: peak power of 175 W, duty cycle of 10%, 200 cycles per burst, and duration of 480 s. Fragmented DNA was purified using SPRISelect beads (Beckman Coulter) using a 1:0.6 ratio of sample to beads to remove high mass fragments above ~300 bp. Concentration of fragments were measured with the Qubit dsDNA HS assay (Thermo Fisher Scientific).

The fragmented samples (i.e., probe set) were mixed to generate a pool, or probe set. An equal weight mixture of the three TFs probes was prepared. The final concentration of the probe set was adjusted to 0.5 ng/µl.

For the following gene sets, OCS probes were synthesized using the same protocol as for the three TFs, with exceptions described below.

Using a protocol similar to that used for synthesis of the three TFs probeset, an OCS probe set was created which correspond to ORFs from the External RNA Controls Consortium, or ERCC. Differences in the protocol are described in the following section. The ERCC has compiled a collection of 92 synthetic ORF sequences, in the form of plasmids, from which RNA standards have been prepared by various vendors. We obtained the ERCC DNA Sequence Library for External RNA Controls (SRM 2374, NIST), a collection of all ERCC ORFs in the pT7T318 vector.

ERCC ORF inserts were PCR amplified with hot-start KOD polymerase (Invitrogen) using M13 primers, sequences below:

M13_canon_FOR: GTAAAACGACGGCCAGT

M13_canon_REV: CAGGAAACAGCTATGAC

PCR was performed for 18 cycles, with each cycle consisting of denaturation at 94 °C for 30 s, annealing at 55 °C for 30 s, and extension at 72 °C for 5 min. The final extension was for 15 min.

An amplicon pool was prepared for 64 ERCC ORFs (Supplementary Table 2). To make the ERCC64 amplicon pool, PCR reactions were pooled with adjustment based on length of the ORF, where higher volumes were used for longer ORFs. Using the pool of amplicons as template, a single biotin-labeling PCR was done. The final probe set is designated as *ERCC64* in this manuscript.

Using a protocol similar to that used for synthesis of the three TFs probeset, an OCS probe set was created which correspond to ORFs from the Spike-in RNA Variant Control Mixes, or SIRV Mixes (Lexogen). The probe set is designated as *SIRV7* in the manuscript. Differences in the protocol are described in this section. The SIRV Mixes are 69 synthetic transcripts from seven genes which mimic the highly complex splicing patterns found in the human transcriptome^33^. We obtained PCR products of SIRV constructs corresponding to SIRV101, SIRV201, SIRV301, SIRV403, SIRV510, SIRV601, and SIRV701. ORF-specific primers were used for the biotin-labeling PCR and were designed to anchor the ATG-start and just upstream of the stop codon. All SIRV primer sequences used may be found in Supplementary Table 3.

Using a protocol similar to that used for synthesis of the three TFs probeset, OCS probe sets were created from different size pools of ORFs derived from transcription factor (TF) genes. For example, *TF2* is a probe set that corresponds to two TFs. The probe sets are designated as *TF2, TF12, TF88, TF682, TF763* in the manuscript. These probe sets were synthesized for the purpose of enriching transcripts from different sets of human transcription factors (TFs). To make pools, ORF clones in the form of bacterial stocks were cherry-picked from the human ORFeome collection using Genesis Automated Liquid Handler (Tecan). PCR success was checked by running a subset of the reactions on an E-Gel 96 agarose gel (Thermo Fisher Scientific). Pools corresponding to 2, 12, 88, and 682 ORF amplicons were used as template for biotin-labeling PCR. For TF763, several pools were made, each containing ORFs of a similar length. Each pool underwent a separate biotin-labeling PCR. TFs belonging to each probe set may be found in Supplementary Table 4.

### Sequence validation of OCS probes

Each OCS probe set was subjected to Illumina sequencing to verify the probe identities and abundances across the source templates. The Kapa DNA Hyper prep kit (Roche) was used, in which barcoded Illumina adapters were ligated directly to the probes. Samples were prepared on a Beckman Coulter Biomek FX. For each sample, approximately 50,000 paired-end reads of length 150 bp were generated on an Illumina MiSeq.

To estimate the representation of each ORF within a given probe set, Kallisto^31^ (version 0.44.0, default parameters) was used to estimate gene-level abundances. Paired-end reads were analyzed. Alignment indices were prepared from a FASTA file containing all human ORFeome sequences. For analysis of probe sets involving ERCC or SIRV standards, the relevant sequences were included in the FASTA file.

To generate read coverage plots across the ORF, reads were first aligned to the human ORFeome using Bowtie2^53^ (version 2.2.3) using “—local” option with default parameters. The alignment file, in SAM format, was parsed using SAMtools^54^ (version 1.2) and custom Python scripts were used to extract read coverage across the ORF on a per-nucleotide basis.

### Commercial probe synthesis

IDT Lockdown probes (Integrated DNA Technologies) were designed and synthesized for *ARNTL*, *STAT1*, and *ZNF268* (Supplementary Table 1). The probes are high purity oligonucleotides (120-mers) with a biotin conjugated at the 5’ end. For each target ORF, a ~1X tiling density was maintained by designing probes that randomly tile the forward and reverse complement sequences. Following reconstitution to 0.75 pmol/µl with TE buffer, probes from the targets were combined in equimolar ratios.

For the single probe enrichments, *ARNTL* probes “ARNTL_forward_7” (returned 8% on-target) and “ARNTL_revcomp_17” (returned 10% on-target) were used. See Supplemental Table 1 for sequences.

### Quantitative PCR (qPCR) method development

Standard solutions of ORF inserts from *ARNTL*, *STAT1*, *ZNF268*, *MBP*, and *HSP90AB1* were prepared for use in absolute quantification and qPCR method validation. These standards comprise purified ORF amplicons in which the absolute concentration of the amplicon is known. ORF inserts were amplified from Gateway clones using M13 primers and Platinum Taq DNA Polymerase High Fidelity, as described earlier. Products were purified with 1.8× volume of Ampure XP beads and amplicons were run on an agarose gel to confirm presence of a single band of expected size. Final concentrations were measured by the Qubit dsDNA HS assay (Invitrogen) and Nanodrop spectrophotometry (Thermo Fisher Scientific). Molarity of each amplicon was calculated based on their sequence and concentration, accounting for vector backbone.

A qPCR method was developed to allow for quantification of target (three TFs) and background (from high abundance housekeeping genes) ORFs (see Fig. 1). TaqMan PCR assays (Integrated DNA Technologies) were designed against 450–500 bp regions within each of the five genes. The long target region length enables quantification of full-length target cDNAs with minimized background interference from the OCS or IDT oligonucleotide probes. The PrimeTime Gene Expression Master Mix and accompanying protocol was used as per manufacturer’s protocols, except for the extension time, which was increased to 120 s. Because of the unconventionally long qPCR target length, we performed full validation of the qPCR method and established excellent linearity (*R*^2^ = 1.00), precision (0.73–1.13% CV), and limit of detection (3.2e-16 to 3.2e-10 M) for each of the five genes.

### Preparation of cDNA

cDNA was prepared using the SMARTer cDNA synthesis kit (Clontech). Approximately 1 µg of total RNA was input per reaction. The brain total RNA was obtained from Biochain and the total RNA of 7 tissues was obtained from Ambion. The manufacturer’s protocol was followed, except for the use of a custom oligo(dT)_30_ containing a 16-mer barcode at the 5’ end, thereby uniquely labeling each cDNA preparation (Supplementary Table 12). After cDNA synthesis, whole cDNA amplification was performed so as to generate sufficient cDNA for multiple capture reactions. The number of PCR cycles was optimized so as to avoid overamplification; this was done by monitoring product formation in small-scale PCR reactions and examining the product size and purity by agarose gel electrophoresis.

### Preparation of spike-in mixtures

An RNA mixture was prepared in which the ERCC standards were spiked into human tissue RNA. Spike-in mixtures were prepared in which 1 µl of a 1:10, 1:80, or 1:5120 dilution of ERCC RNA Spike-In Mix (mix 1, Thermo Fisher) were each combined with 1 µg of UHRR (Biochain). Each mixture was converted to cDNA as in the section “Preparation of cDNA.”

An RNA mixture was prepared in which the SIRV standards were spiked into human tissue RNA. Spike-in mixtures were prepared in which 2.5 µl of a 1:10 dilution of SIRV RNA Spike-In Mix (mix E0, Lexogen) were each combined with 1 µg of UHRR (Biochain). Each mixture was converted to cDNA as in the section “Preparation of cDNA.”

### Full-length cDNA enrichment

This protocol was adopted from the following two protocols: (i) “Hybridization capture of DNA libraries using xGen Lockdown Probes and Reagents” from IDT (version 2) and (ii) “cDNA Capture Using IDT xGen Lockdown Probes” from PacBio (Part Number 101-604-300 Version 01).

For every capture experiment, an adequate amount of cDNA was prepared from RNA or mixtures of RNA. Approximately 1 µg of purified cDNA was combined with 1 nmol of Clontech primer and 1 nmol of oligo(dT)_18_ containing a three-carbon spacer at the 3’ end (Eurofins Scientific), oligonucleotides that serve as blockers. The solution was dried down using vacuum centrifugation and subsequently resuspended in 8.5 µl 2× hybridization buffer, 2.7 µl enhancer buffer, and 1.8 µl of water, reagents supplied from the IDT Lockdown xGen kit.

Hybridization experiments, in which the probes are allowed to bind to the target cDNA, were performed using the following conditions. The cDNA was heated to 95 °C for 10 minutes, followed by a rapid ramp down to 65 °C. Either 4 ng of OCS probes or 3 pmol of IDT probes were added and the mixture was incubated at 65 °C for 4 hours. 50 μl of M-270 streptavidin beads (Invitrogen) were added and a series of washes were performed according to the IDT xGen Lockdown protocol version 2, except that initial washes used wash buffer pre-heated to 72 °C instead of 65 °C to reduce non-specific binding.

Following hybridization-based capture, an on-bead PCR was performed to generate adequate amounts of captured material for sequencing. After the washes, the final bead solution was resuspended in 50 µl of TE buffer. To amplify the full-length cDNAs that were captured on the beads, on-bead PCR was performed with 5 µl of resuspended beads in a 30 µl reaction using KAPA HiFi HotStart 2X mix (KAPA) and the universal Clontech primer. The program was run for 30 cycles, with each cycle consisting of denaturation at 98 °C for 20 s, annealing at 65 °C for 15 s, and extension at 72 °C for 5 min. The final extension was for 10 min.

In some cases, rather than an on-bead PCR, a heat elution was performed for the purpose of quantifying abundances of bound cDNA via qPCR. An aliquot of beads was diluted 10-fold with buffer EB (10 mM Tris-HCl, pH 8.0) (Qiagen) and heated at 95 °C for 5 min. Beads were placed on a magnet and supernatant recovered for subsequent qPCR analysis.

### Enrichment of TFs from cDNA from 7 human tissues

A capture experiment was performed using TF763 against the pool of cDNA from seven human tissues. Since the PacBio technology employs diffusion-based loading of SMRTBell libraries, and because shorter cDNA molecules tend to more efficiently diffuse into the sequencing nanowells, an inherent bias against longer cDNAs is observed. Therefore, to increase the recovery of transcripts across longer lengths, a second capture was performed to increase recovery of longer transcripts. The seven tissue-specific cDNAs were size selected using SPRISelect (Beckman Coulter) so that only transcripts above ~2 kb were recovered. A second capture was performed using TF763 against the 2 kb+ size-selected cDNA. The capturant involving the full-size cDNA and the capturant involving the 2 kb+ size-selected cDNA were each sequenced on a 1 M SMRTcell on the PacBio Sequel system. Therefore, a total of two 1 M SMRTcells were run for the capturant. The original, unenriched input cDNA was also sequenced on an independent 1 M SMRTcell.

### Illumina library preparation and analysis

The transcript abundances in human brain RNA were quantified through RNA-Seq (data used in Fig. 1). cDNA was synthesized from total RNA from the cortex region of human brain (Biochain) using the protocol described in Preparation of cDNA. cDNA was converted into an Illumina library using the NEBNext protocol (New England Biosciences) and ~20 million PE75 reads were sequenced on an Illumina NextSeq, in duplicate. Sequencing data was collected at the Center for Cancer Computational Biology (CCCB) at the Dana-Farber Cancer Institute.

To estimate expression values for each gene, RSEM was used with the STAR aligner. The STAR genome index was built based on hg38 and using annotation obtained from GENCODE (version 27). Transcripts per million (TPM) values were calculated using RSEM (version 1.2.29)^55^.

The following section describes the sequencing and quantification of enriched cDNA from capture experiments. Sequencing data was collected for the enrichment of three TFs (Fig. 1d), ERCC ORFs (Fig. 2b), and the other TFs studied (Fig. 4a). Illumina sequencing data was collected following the procedure described in Sequence validation of OCS probes. To quantify isoform- and gene-level expression, Kallisto (version 0.44.0) was used using default parameters. To estimate values for all human genes (as in Figs. 1d, 4a), Kallisto indices based on GENCODE (version 27) transcript sequences were used. To estimate expression values for each ERCC ORF, Kallisto indices based on ERCC and GENCODE (version 27) sequences were used.

For the TF multiplexing experiment described in Fig. 3, Illumina sequencing data was collected on cDNA subjected to a workflow similar to Nextera sequencing (Plexwell sequencing, SeqWell). For gene quantification, Kallisto (version 0.44.0) was used with default parameters.

For orthogonal validation of TF isoforms (see Fig. 4), the following RNA-Seq protocol was performed. Human tissue total RNA samples were converted to Illumina libraries using the KAPA mRNA Hyper Prep kit, as per manufacturer’s protocol (KAPA). Libraries were barcoded using TruSeq Illumina Adapters Sets A and B (Illumina).

### PacBio library preparation

For each reaction, ~1 µg of either input cDNA or captured cDNA was converted into a SMRTbell library using the SMRTbell Template Prep Kit 1.0 (Pacific Biosciences) and sequenced on either a PacBio RSII or Sequel I (Pacific Biosciences).

### PacBio data analysis with Iso-Seq 3

Bioinformatics analysis was done by using the Iso-Seq 3 application in the PacBio SMRT Analysis v6.0 to obtain high-quality, full-length transcript sequences, followed by downstream analysis, as described below.

First, full-length reads were identified from the raw data. Full-length reads were determined as circular consensus sequence (CCS) reads that contained both the 5′ and 3′ primer and the polyA tail preceding the 3′ primer. The 5′ primer consists of the Clontech SMARTer cDNA primer with an ATGGG overhang. The 3′ primer consists of a 16 bp-long PacBio barcode that is sample-specific followed by the Clontech SMARTer cDNA primer.

Second, isoform-level clustering was done to obtain high-quality transcript sequences. To increase detection of rare isoforms, the de-multiplexed full-length reads were pooled to perform isoform-level clustering analysis^38^. After clustering, consensus sequences were called using the Arrow algorithm (within SMRT Link version 7 software) and only polished sequences with predicted consensus accuracy ≥99% were considered high-quality and retained for the next step.

Third, clustered sequences were mapped to the reference genome and filtered for on-target isoforms. The high quality transcript sequences were mapped to hg38 using minimap2^56^ (version 2.11-r797) using parameters “-ax splice -t 30 -uf --secondary=no -C5”. We then filtered transcripts mapped to targeted probe region with ≥ 99% coverage and ≥ 95% identity.

Fourth, the final isoforms were de-multiplexed by sample barcodes. We recovered the relative abundance of each of the final isoforms in each sample by extracting the fraction of full-length reads supporting each isoform from each sample.

Last, isoforms were annotated and assessed for quality with SQANTI2. SQANTI is a computational tool for annotation and quality assessment of full-length isoforms sequenced on long-read platforms^39^. We adapted SQANTI so that it includes additional functional and quality features relevant to isoform quality, a version called SQANTI2 (unpublished, github: https://github.com/Magdoll/SQANTI2). All de-multiplexed isoforms from the Iso-Seq 3 pipeline was processed with SQANTI2 using default parameters. Isoform and junction annotation and feature files, including match information to GENCODE version 29, were output.

### Isoform overlap estimation

GENCODE v29 human annotations were parsed to determine the overlap between the reference isoform and all other isoforms of that gene. First, a reference isoform, as defined as the principle isoform in APPRIS, was defined for each protein-coding gene^34^. The exon-intron structures for each APPRIS isoform was compared to the exon-intron structure of every isoform of that gene using bedtools intersect function (v2.27.1). We considered isoforms as overlapping if the number of overlapping base pairs was 50 bp or higher. Only genes and isoforms which were annotated as protein-coding were considered in this analysis. Only transcripts with the GENCODE “basic” tag (i.e., core GENCODE set, not the comprehensive set) was considered for this analysis. A similar analysis was done as described, but with a randomly chosen GENCODE isoform. SIRV isoform overlap was calculated using a similar routine.

### Saturation-discovery curves

For the TF multiplexing experiment, saturation-discovery curves were generated by subsampling the pool of full-length reads at different depths. Full-length reads were drawn at random and, for each subsampled pool of reads, the number of unique genes or transcript isoforms detected was determined. For each sampling depth, 100 iterations of sampling were done, and the average of the number of unique genes or isoforms observed was computed. Only isoforms exactly matching GENCODE (v29) were considered. The saturation-discovery curve analysis uses ‘subsample.py‘ and ‘subsample_with_category.py‘ scripts in https://github.com/Magdoll/cDNA_Cupcake.

### GENCODE recoveries

For each GENCODE isoform (i.e., ENST) in version 29, we determined if there was an exact match in the PacBio transcripts. An exact match is defined as cases in which the detected full-length isoform contains an exact sequence of junctions (i.e., introns) as found in the GENCODE transcript. This was accomplished using a modified version of the SQANTI program, SQANTI2.

### Functional features of isoforms annotated within SQANTI2

In this section is described the various functional features assessed within SQANTI2 to evaluate the quality of novel isoforms, which includes CAGE peak overlap, junction conservation, and polyA motifs.

For the CAGE peak overlap, the overlap between the 5′ end of isoforms and CAGE peak data was assessed. CAGE peak annotations^57^ were downloaded from: http://fantom.gsc.riken.jp/5/datafiles/latest/extra/CAGE_peaks/hg19.cage_peak_phase1and2combined_coord.bed.gz

Genomic coordinates were converted from hg19 to hg38 using the liftOver program from the UCSC Genome Browser^58^. The genomic position of the 5′ end of the isoforms was compared to all CAGE peaks and the following properties were described: (i) the distance between the 5′ end and the center position of the closest CAGE peaks, and (ii) whether the 5′ resided within a range of a CAGE peak.

The conservation at each junction was obtained through downloading phyloP scores for each nucleotide in the human genome (hg38)^59^. PhyloP conservation scores for each donor and acceptor site were obtained. The dinucleotides at the splice donor (e.g., GT) as well as the adjacent nucleotide residing on the exon were analyzed for the 5′ splice site. The dinucleotides at the splice acceptor (e.g., AG) as well as the adjacent nucleotide residing on the exon were analyzed for the 3′ splice site. Therefore, a trinucleotide was analyzed for each splice site.

A polyA motif is commonly found upstream of the site of cleavage and polyadenylation. The highest frequency polyA motifs in human are AAUAAA and AUUAAA, considered canonical motifs due to their high frequency^60^. The genomic position of the 3′ end of the isoforms was located, and it was determined whether there was presence of a canonical motif 5–25 nucleotides upstream of the 3′ site.

### PacBio data analysis for chimera rate estimations

The data analysis of the barcoded cDNA material for estimation of non-chimeric isoforms is similar to that published previously^40^. The data was analyzed by generating CCS reads using the ‘ccs‘ algorithm in SMRT Link 8.0 with parameters ‘--min-rq 0.8 –min-passes 1‘. Then, the demultiplexing tool ‘lima‘ (version 1.11.0 in smrttools/incremental, Jan-2020) was used in two different modes to identify and remove the sample barcodes, cDNA primers, and concordant barcode pairings. In the first ‘lima‘ iteration, the multiplexed sample barcodes were provided which contained the PacBio 16 bp barcodes and the Clontech primers. This iteration of ‘lima‘ with parameters ‘ --dump-clips --split-bam-named –same‘. In the second ‘lima‘ iteration, the 96 pairs of dual barcodes were provided, where the expected 5’ end contained both the 16 bp barcode from IDT and the SP6 sequence (5′-CATACGATTTAGGTGACACTATAGG-3′). In this second iteration, ‘lima‘ was run using the parameters ‘--isoseq --dump-clips‘.

When run with ‘--isoseq‘ mode, ‘lima‘ finds for each sequence the highest scoring pairing between a given 5′ and 3′ primer. Thus, concordant cDNA molecules would have matching 5’ and 3’ primers in the ‘lima‘ output. Conversely, a discordant molecule from a PCR reaction would have mis-matched primer pairings.

Finally, all full-length reads from all samples, including both concordant and discordant reads, were pooled together to run through ‘isoseq3 cluster‘ and ‘isoseq3 polish‘ step to get high-quality (HQ) isoforms. The HQ isoforms were then mapped to hg38 and collapsed using the Cupcake tool (https://github.com/Magdoll/cDNA_Cupcake) with 99% coverage and 95% identity cutoff. The collapsed results were then demultiplexed to get per-sample full-length read counts. Isoforms were determined to be concordant if a majority of their full-length reads arose from non-chimeric reads. SQANTI2 was run with GENCODE v29 as the reference annotation.

### Reporting summary

Further information on research design is available in the Nature Research Reporting Summary linked to this article.

## Supplementary information

Supplementary Information

Description of Additional Supplementary Files

Reporting Summary

Supplementary Data 1

Supplementary Data 2

Supplementary Data 3

Supplementary Data 4

Supplementary Data 5

Supplementary Data 6

Supplementary Data 7

Supplementary Data 8

Supplementary Data 9

Supplementary Data 10

Supplementary Data 11

Supplementary Data 12

## Data Availability

The data supporting the findings from this study are included either in the manuscript or its associated supplementary files. Sequencing data has been deposited to the Sequence Read Archive (SRA) and can be found under BioProject PRJNA615244. The processed PacBio sequencing data (SQANTI2 output) have been deposited to the Zenodo database (https://zenodo.org). All data are also available from the authors upon request. Data underlying the figures and supplementary figures are included in the Source Data tables. The CAGE peak data, which is publicly available from the FANTOM consortium, may be found using the following link: http://fantom.gsc.riken.jp/5/datafiles/latest/extra/CAGE_peaks/hg19.cage_peak_phase1and2combined_coord.bed.gz

## Source Data

Source Data
