# Supplementary material for: ORF Capture-Seq as a versatile method for targeted identification of full-length isoforms
Source: Nat Commun. 2020 May 11;11:2326. doi: 10.1038/s41467-020-16174-z (PMC7214433; doi:10.1038/s41467-020-16174-z)
Supplement: Supplementary file 1 — Supplementary Information [file 41467_2020_16174_MOESM1_ESM.pdf]

Supplementary Information

ORF Capture-Seq as a versatile method for targeted identification of full-length isoforms

Sheynkman *et al*.

Supplementary Figure 1

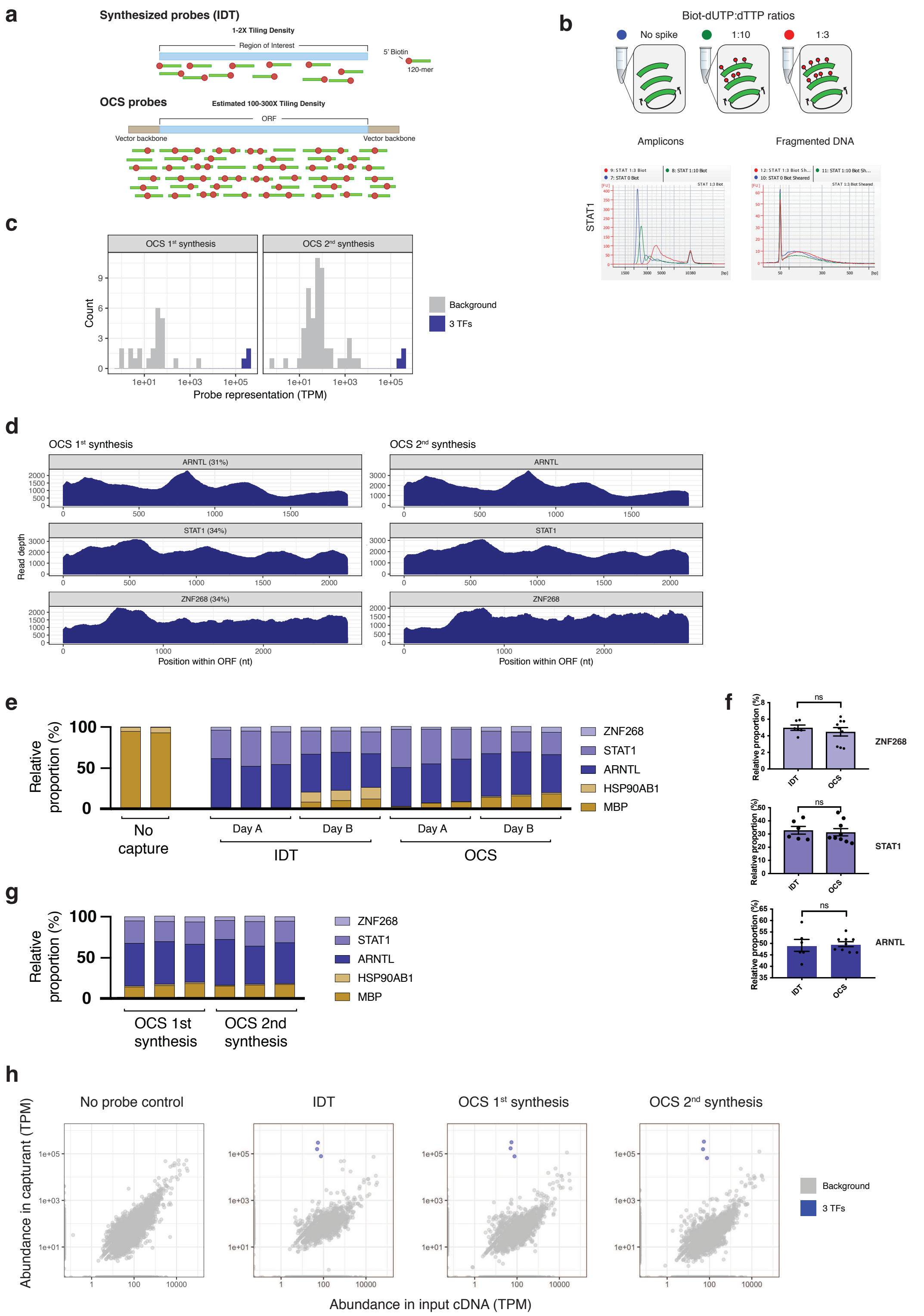

Supplementary Figure 1 OCS and IDT probe sets perform comparably. a Schematic of commercially synthesized (IDT) probes and OCS probes. b QC of biotin-dUTP-labeling PCR and fragmentation. Biotin-dUTP replaced dTTP in the PCR mix at ratios of 1:10 and 1:3. The lower panel shows Bioanalyzer traces of the amplicons before and after sonication (Methods). Color of legend dots and plotted lines (blue, green, red) correspond to different levels of biotin-dUTP spikes. c Purity of probe sets. Abundance of probes from on-target (dark blue) versus off-target (gray) ORFs. d Probe coverage across the source ORF templates. Read depth is the number of aligned reads at each nucleotide position. Reads were sequenced on an Illumina MiSeq (Methods). e Full dataset for the comparison of IDT vs OCS-based target enrichment. Each bar shows the relative proportion of cDNA from target (purple) versus background (yellow) genes as quantified by qPCR (average of two technical replicates). Three individual capture reactions were performed per day over two days (Day A, B). f Comparison of on-target proportions between IDT and OCS probes. P-values were calculated using the Mann-Whitney two-tailed test. Each replicate represents an independently executed capture reaction. Error bars, s.e.m. (IDT, n=6; OCS, n=9; all data from independent experiments); n.s., not significant (P-value is above 0.05). g Comparison of capture experiments using two independent syntheses of OCS probes. h Background binding profiles. Comparison of gene abundance of input and capturant for each experiment. TPM, transcripts per million.

Supplementary Figure 2

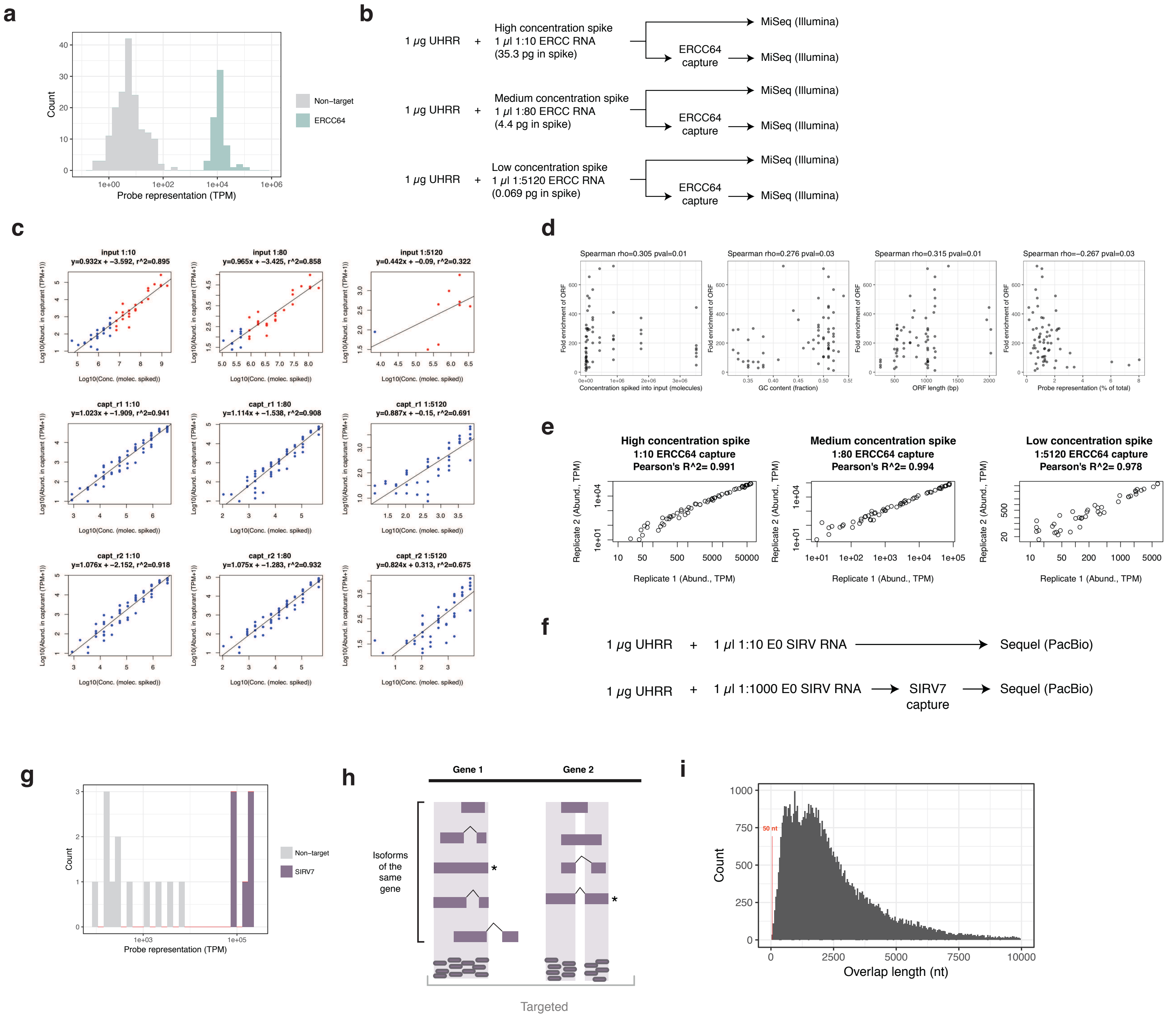

Supplementary Figure 2 Benchmarking OCS analytical performance with spike-in standards. a Purity of ERCC64 probe set. Plots show abundance of probes derived from targeted ERCC templates (light blue) versus non-targeted genes (gray). b Schematic of ERCC spike-in capture experiment. 1:10, 1:80, and 1:5120 are 10-, 80-, and 5120-fold dilutions of the ERCC spike-in mix 1. These correspond to the high, medium, and low spike, respectively. ERCC, External RNA Controls Consortium; UHRR, universal human RNA reference. c Linearity of ERCC standards, before and after capture. Linear regression performed on all 92 ERCC ORFs for input (top panel), and 64 targeted ERCC ORFs in the capturants (middle and bottom panels). Data for two independent captures (rep1, rep2) are shown. Equation of best fit line and R2 is shown for each plot. d Plots showing the relationship between ERCC ORF properties and enrichment efficiency. Spearman's rho and associated p-value shown. e Reproducibility of technical replicates of ERCC64 captures. Pearson's correlation calculated for the 64 ERCC ORFs. f Purity of SIRV7 probe set. Plots show abundance of probes derived from targeted SIRV templates (purple) versus non-targeted genes (gray). Note that one SIRV per locus was selected to be included in the probe set. g Schematic of the SIRV experiment. Isoforms with an asterisk mark the representative SIRV selected for each locus. h Schematic of the SIRV capture experiment. SIRV, Spike-in RNA Variant Control; UHRR, universal human RNA reference. i Distribution of overlap lengths between the GENCODE principal isoform and all isoforms of that gene. GENCODE version 29 was used in this analysis. The position of 50 nt is marked by the red line, denoting the threshold under which isoform enrichment efficiency is expected to marked decrease.

Supplementary Figure 3

a

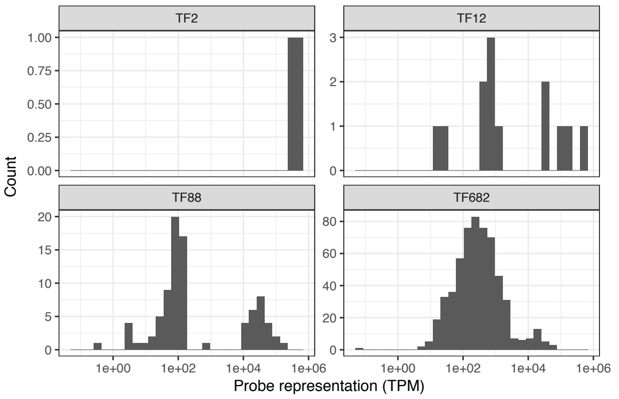

b

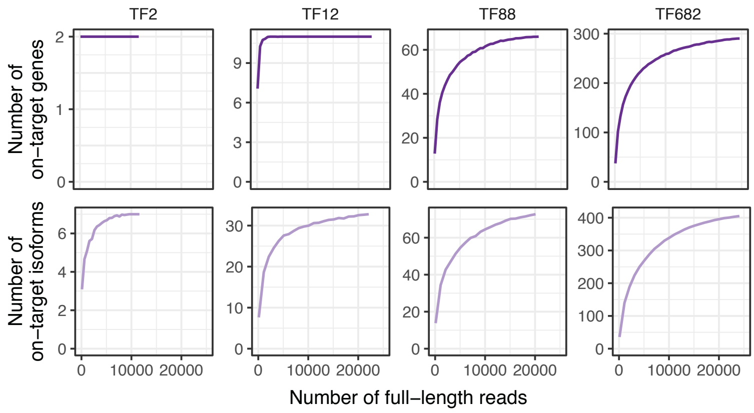

Supplementary Figure 3 Multiplexing OCS captures. a Distribution of the abundances of probes, on a per-ORF-basis. Results for TF2, TF12, TF88, and TF682 are shown. X-axis shows transcripts per million, which was calculated per ORF. b Saturation-discovery curves for the number of detected genes (dark purple) and isoforms (light purple). Full-length reads were subsampled without replacement from the original data 100 times. For each population of samplings, the average number of genes or known isoforms recovered was calculated.

Supplementary Figure 4

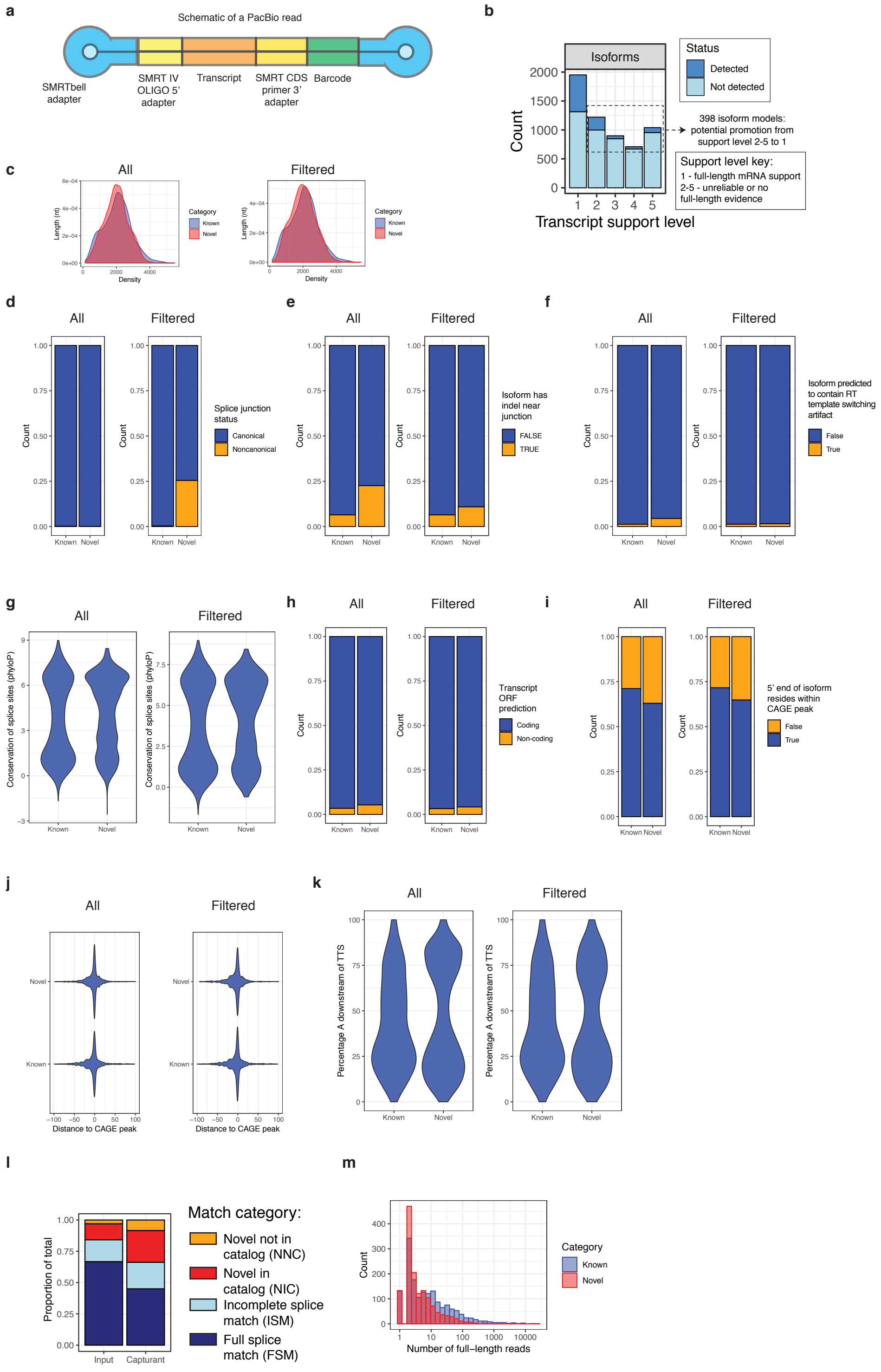

Supplementary Figure 4 Enrichment and characterization of novel TF isoforms. a Depiction of structure of final PacBio reads after library preparation. b Full-length sequence data applied towards validation of existing gene models. Plot shows the number of GENCODE-annotated isoforms for each "transcript support level " (i.e., extent of empirical evidence underlying an isoform). Data shown only for the genes targeted in the TF enrichment experiment (TF763). Fraction of all isoforms that exactly matches, and thus is validated by a full-length sequenced transcript, is shown in dark blue. c-k Comparison of sequence and functional features between known and novel isoforms, before and after filtering based on Illumina data. Comparisons include c distribution of the transcript length; proportion of isoforms containing d non-canonical junctions, e indel sequencing errors adjacent to the splice junction, and f a predicted reverse transcription template switching artifact; g distribution of phyloP-based conservation at nucleotides residing adjacent to the junction (Methods); proportion of isoforms which h are predicted as containing a coding ORF (SQANTI, GMST, see Methods), and i contain a 5' end residing within a CAGE peak (Methods); j distribution of distances between 5' end of isoform and an annotated CAGE peak, and k distribution of the percentage of A/T content, on the genome, which is immediately downstream of the 3' site as detected by the sequenced isoform. l Proportions of known and novel isoforms, plot similar to Figure 4e, except proportions are normalized to 1. Known isoforms are further divided by completeness. Novel isoforms are further divided by whether all splice sites are found in GENCODE (novel in catalog, NIC) or if the isoform contains a novel splice site (novel not in catalog, NNC). Match categories are defined by the isoform annotation tool SQANTI. m Distribution of full-length read depth for known and novel isoforms.
