# Supplementary material for: ORF Capture-Seq as a versatile method for targeted identification of full-length isoforms
Source: Nat Commun. 2020 May 11;11:2326. doi: 10.1038/s41467-020-16174-z (PMC7214433; doi:10.1038/s41467-020-16174-z)
Supplement: Supplementary file 2 — Description of Additional Supplementary Files [file 41467_2020_16174_MOESM2_ESM.docx]

**Description of Additional Supplementary Files**

**File name: Supplementary Data 1**

**Description:** Sequences of biotinylated oligos synthesized for capture of TFs in Figure 1.

**File name: Supplementary Data 2**

**Description:** List of ERCC ORFs belonging to probe set *ERCC64*.

**File name: Supplementary Data 3**

**Description:** Primers used to amplify SIRVs for probe synthesis.

**File name: Supplementary Data 4**

**Description:** List of genes targeted in the TF multiplexing experiment. Includes the ORF sequences used as template for probe synthesis.

**File name: Supplementary Data 5**

**Description:** Abundance of probes within each probe set in the TF multiplexing experiment. Units are in transcripts per million (TPM).

**File name: Supplementary Data 6**

**Description:** Variability in probe abundance within each probe set in the TF multiplexing experiment. Summary statistics are based on distribution ORF-specific TPMs.

**File name: Supplementary Data 7**

**Description:** List of genes targeted in the TF enrichment experiment.

**File name: Supplementary Data 8**

**Description:** Abundance of probes within each probe set in the TF enrichment experiment. Units are in transcripts per million (TPM).

**File name: Supplementary Data 9**

**Description:** Proportion of GENCODE genes and isoforms detected in the TF enrichment experiment.

**File name: Supplementary Data 10**

**Description:** GENCODE isoforms, categorized by their transcript support levels (TSLs), detected in the TF enrichment experiment.

**File name: Supplementary Data 11**

**Description:** High-quality isoform sequences detected in the TF enrichment experiment. Match category, as defined within the SQANTI program, is listed for each isoform.

**File name: Supplementary Data 12**

**Description:** Oligo(dT) barcode sequences used in first strand synthesis during cDNA preparation.
